# How physical exercise with others and prioritizing positivity contribute to (work) wellbeing: a cross-sectional and diary multilevel study

**DOI:** 10.3389/fspor.2024.1437974

**Published:** 2024-09-06

**Authors:** Ester Gil-Beltrán, Cristian Coo, Isabella Meneghel, Susana Llorens, Marisa Salanova

**Affiliations:** ^1^ WANT Research Team, Universitat Jaume I, Castellón, Spain; ^2^ Mind AS, Trondheim, Norway; ^3^ Area of Psychology and Mental Health, Universidad Internacional de Cataluña, Barcelona, Spain

**Keywords:** physical exercise, engagement, positive emotion, prioritizing positivity, upward spiral theory of lifestyle change

## Abstract

**Introduction:**

This work is a dual study employing a cross-sectional approach and a diary method to investigate how physical exercise can become a habit. Guided by the Upward Spiral Theory of Lifestyle Change, we examined the role of prioritizing positivity and engaging in physical exercise with others as advantageous resources and their impact on the relational loop of physical exercise behavior, emotions, and engagement.

**Methods:**

The first study involved a sample of 553 participants, and the second study included 146 participants, all of whom were employed and regularly engaged in physical exercise. We utilized structural equation modeling and multilevel analysis for the respective studies.

**Results:**

The results of the first study indicate that individuals exercise more when they experience higher levels of engagement and positive emotions, particularly when exercising with others and prioritizing positivity. The findings of the second study reveal that prioritizing positivity acts as a precursor to positive emotions during physical exercise, which in turn reinforces the relational loop between emotions and exercise behavior.

**Discussion:**

Both studies conclude that individuals who prioritize positivity experience better psychological wellbeing and higher engagement in physical exercise.

## 1 Introduction

One of the growing lifestyle challenges in today's world is sedentary behavior. Work and free time are increasingly related to technology, consequently leading people of all ages to spend more time interacting with technology in the form of the Internet, video games, interactive television, mobile phones, and other platforms; also, commuting from home to work and vice versa is done frequently by car, further limiting physical activity. Due to the pandemic, technologies have become even more crucial, with teleworking, digital socializing, and online hobbies becoming integral parts of our lives. This results in the average adult spending more than half of their day sedentary, contributing to an increase in sedentary lifestyles in industrialized countries over recent decades (1). Specifically, Eurobarometer studies between 2005 and 2017 have shown an increment in the prevalence of sedentarism among adults, with the increment being higher in men than in women (2). This concern about sedentary lifestyles stems from different studies indicating that people who accumulate more than 4 h of sedentarism every day face an increased risk of suffering from cardiovascular diseases and premature death (3, 4), which makes sedentary lifestyle an important risk factor.

Already at the beginning of the 21st century, large world organizations proposed physical activity as a palliative for sedentary lifestyles; for instance, the World Health Organization introduced the Physical Activity and Health in Europe report (5), the EU released the Physical Activity Guidelines (6), and the United States published the Physical Activity and Health Report (7). These guides are based on the fact that leading an active life not only provides numerous physical health benefits but also enhances social and psychological wellbeing.

How can we ensure that physical exercise (PE) becomes a lasting habit rather than a temporary fad? Some studies suggest that behaviors associated with enjoyment are more likely to be sustained; that is, behaviors such as performing PE are more likely to be repeated in the future if they are considered pleasant rather than merely beneficial (8). This highlights the role of the affective part of the behavior in fostering consistent behavior, leading to greater benefits and creating a positive vicious circle.

A theoretical explanation of this fact is given by the Upward Spiral Theory of Lifestyle Change (9). This theory consists of two loops, which are based on two theories: (1) the incentive salience theory of addiction (10, 11) and (2) the broaden-and-build theory of positive emotions (12–14). The first loop, explained by the incentive salience theory, tells us how the positive affect that we experience when adopting a behavior creates unconscious motives associated with the signals that the behavior is going to occur, and over time, these unconscious motives strengthen the decision to persist in this behavior. The second loop, explained by the broaden-and-build theory of positive emotions, tells us how, over time, repeated exposure to positive affect creates the so-called vantage resources, which strengthen the relationship between behavior and positive affect. These vantage resources can be biological (vagus nerve or the oxytocin system), social (social support), or psychological (prioritizing positivity) (14).

The added value of the current study lies in testing some of the mechanisms that convert a behavior into a habit, following the Upward Spiral Theory of Lifestyle Change (9). Following the principles of the above-mentioned theories, the vantage resources taken into account are (i) performing PE with other people and (ii) prioritizing positivity, as exemplified by how individuals make decisions in organizing their daily lives to make themselves happier. The innovative idea is that when people are doing PE with others and prioritize positivity, the frequency and intensity of PE increase because they are feeling well psychologically (i.e., engagement and emotions related to PE). We examined this wellbeing that is experienced from two aspects: the hedonic aspect, which includes satisfaction with life and affective components (PE emotions), and the eudaimonic aspect, which focuses on optimal psychological functioning (PE engagement) (15).

To achieve our purpose, we used two complementary approaches. On the one hand, we tested the mechanism using a between-subjects design through a cross-sectional study (Study 1). The objective of this first study was to verify the mediation of psychological wellbeing (i.e., PE engagement and PE emotion) in the relationship between vantage resources (i.e., prioritizing positivity and doing PE with others) and performing PE. That is, we investigated whether individuals who prioritize positivity and perform PE with others experience more engagement and affect during PE and thus have an increased habit in PE—performing PE more frequently, for longer durations, and/or at higher intensities.

On the other hand, we tested the mechanism from a within-subject perspective; we supported our research model in the Upward Spiral Theory of Lifestyle Change (9), testing our hypothesis using a diary study (Study 2). The objective of this second study was to observe whether daily variations in the frequency, intensity, and duration of PE sessions are positively associated with the PE-related affect (i.e., PE emotion). In addition, we examined the modulating role of prioritizing positivity as an advantageous resource in the relationship between PE and PE-related affect on that day. We expect that when people prioritize positivity in their life, their sense of effectiveness in physical exercise will be boosted, and this will have a positive effect on their affect at the daily level. Prioritizing positivity each day boosts the sense of control of individuals because they feel in charge of their life agenda. According to Bandura’s Social Cognitive Theory (16), feeling more effective while doing an activity (i.e., physical exercise on a daily basis) leads to more positive feelings and wellbeing.

First, we present the hypotheses, methods, and results of Study 1, followed by the corresponding sections for Study 2.

## 2 Study 1

Study 1 is a between-subject study in which we want to see whether people who prioritize positivity and perform PE with others experience more PE emotions during PE. Also, in turn, we aimed to determine whether these people perform PE more frequently, for longer sessions, and at higher intensities.

Following the Upward Spiral Theory of lifestyle Change (9), the hypothesized model was explored through the following hypotheses and is depicted in Supplementary Figure S1:

○ Hypothesis 1: We expect that vantage resources (i.e., prioritizing positivity and doing PE with others) will be positively associated with psychological wellbeing [i.e., PE engagement (a1, a3) and PE emotion (a2, a4)].
○ Hypothesis 2: We expect that psychological wellbeing [i.e., PE engagement (b1, b2, b3) and PE emotion (b4, b5, b6)] will be positively associated with the practice of PE (i.e., frequency, duration, and intensity).
○ Hypothesis 3: We expect psychological wellbeing [i.e., PE engagement (c1, c2, c3) and PE emotion (c4, c5, c6)] to fully mediate the relationship between vantage resources (i.e., prioritizing positivity and performing PE with others) and the characteristics of the PE (i.e., frequency, duration, and intensity).

### 2.1 Materials and methods: study 1

#### 2.1.1 Participants and protocols

This study was conducted online during the COVID-19 confinement period in 2020 and consisted of two phases. Phase 1 took place during the lockdown period and involved a cross-sectional study, for which a call for participation was launched through social networks, encouraging people to participate in the study. For this, a link giving access to the survey was shared, and 1,266 individuals participated anonymously. The sample for Study 1 was selected from this general sample, by choosing only those individuals who were performing PE during the confinement. It consisted of 553 participants, of which 61% were women, with a mean age of 41 years (SD = 10.62), and 77% of them were working from home.

In this phase, the participants were asked if they wanted to participate in Phase 2, from which the sample for the second study would be drawn.

#### 2.1.2 Measures

The variables and questionnaires used for the study are described as follows:

• Prioritizing positivity: This was evaluated through a six-item scale (17) (*α* = 0.83) (e.g., “A priority for me is experiencing happiness in everyday life”; “I look for and nurture my positive emotions”). It was measured with a Likert-type scale, ranging from 0 (never) to 6 (always).
• PE with others: This was evaluated with a behavioral item that refers to whether, during confinement, they performed PE alone or with others [“Generally, you are doing physical exercise alone (1 = 70.3%) or in company (2 = 29.5%)”].
• PE engagement: This was evaluated using the UWES-3 using three items (18) (*α* = 0.88) but adapted to PE (“When I do physical exercise, I feel full of energy”; “During confinement, I feel excited doing physical exercise”; “During confinement, time flies when I do physical exercise”). It was measured with a Likert-type scale ranging from 0 (never) to 6 (always).
• PE emotions: This was evaluated using a 7-point visual analog scale (19, 20), where a single item asked them to indicate the face that best expressed how they had felt at the level of emotional affect while doing PE (0 = sad face and 6 = happy face) (mean = 5.1; SD = 0.88).
• Physical exercise: This was evaluated using three indicators. First, the frequency with which the participants carried out physical exercise during the week (1–2 days, 3–4 days, 5–6 days, every day, more than once a day); second, the amount of time the participants spent in physical exercise sessions, indicating how long the session lasted (20–30, 31–45, 46–60, 61–90, 91–120, more than 120 min); and third, the intensity with which they did the physical exercise session, which was evaluated using a six-point visual analog scale (19, 20), where a single item asked them to indicate the battery level that best expressed the intensity of their physical exercise sessions (0 = almost empty battery and 5 = full battery) (mean = 2.91; SD = 1.24).

#### 2.1.3 Data analysis

We used IBM SPSS Statistics 26.0 for the descriptive analysis (means, standard deviations), internal consistency analysis (Cronbach's alpha), and internal correlations of the study variables. Also, the common variance bias was checked using the Harman single-factor test (21).

Then, we tested the complete mediation model, including indirect effects, using structural equation modeling [SEM; AMOS 26.0 (IMB Corp., Armonk, NY, USA)]. This allowed us to test all relationships within a single serial mediation model using confidence intervals (22). The mediation of the hypothesized model (Supplementary Figure S1) proposes that wellbeing in PE (PE engagement and PE positive emotions) completely mediates the relationship between vantage resources (prioritizing positivity and PE with others) and performing PE.

### 2.2 Study 1 results

#### 2.2.1 Descriptive analyses and Harman's test

In Supplementary Table S1, the means, standard deviations, and intercorrelations between the study variables can be found. The results show that the PE engagement scale (α = 0.88) and the prioritizing positivity scale (α = 0.83) meet the reliability criteria proposed by previous scientific research (23); the rest of the variables are measure using single items; therefore, reliability cannot be measured. The frequency data of the variables in which we used intervals are PE frequency (1–2 days = 22.8%; 3–4 days = 33.6%; 5–6 days = 22.4%; every day = 18.8%; more than once a day = 2.4%) and PE duration (20–30 min = 25%; 31–45 min = 23.5%; 46–60 min = 36.3%; 61–90 min = 11.9%; 91–120 min = 1.8%; more than 120 min = 1.3%).

The questionnaire consisted mostly of a single item to reduce response time. This is also based on evidence validating the use of single-item scales for assessment (18, 24). The correlation analyses show that the variables are positively related, except for performing PE with others, which did not correlate positively with prioritizing positivity and all of the PE variables (frequency, duration, and intensity).

Second, the results of the Harman test revealed that a single factor explains 38% of the variance. Since it is less than 50%, it can be said that there is no common variance bias (25). Furthermore, the recommendations of Podsakoff et al. (26) were followed, differentiating the different parts of the questionnaire by titles, as well as using different response scales, to minimize the impact of the variance bias of the common method. Therefore, it can be considered that this bias does not affect the study data, so the variance in the variables can be attributed to the evaluated constructs rather than the evaluation method.

#### 2.2.2 Structural equation models

The results of the analyses testing the hypotheses are reported in Supplementary Table S2. Pathways that are central to hypothesis evaluation are depicted in Supplementary Figure S1 and mentioned in Supplementary Table S2 to facilitate readability.

The results fully confirm Hypothesis 1, with significant and positive relationships between prioritizing positivity and both PE engagement (*β* = 0.37, *p* < 0.001) and PE emotions (*β* = 0.27, *p* < 0.001). Similarly, performing PE with others is significantly and positively related to both PE engagement (*β* = 0.29, *p* < 0.01) and PE emotions (*β* = 0.18, *p* < 0.05).

Hypothesis 2 is confirmed only for the relationships between PE engagement and the three PE variables: frequency (*β* = 0.28, *p* < 0.001), intensity (*β* = 0.41, *p* < 0.001), and duration (*β* = 0.48, *p* < 0.01). However, the relationships between PE emotions and PE variables were not significant, except for intensity (*β* = 0.15, *p* < 0.05).

Finally, Hypothesis 3 is also partially confirmed. On the one hand, PE engagement is confirmed as a full mediator in the relationships between vantage resources (prioritizing positivity and PE with others) and PE variables (PE frequency, PE duration, and PE intensity). The indirect effects were all positive and significant, as listed in Supplementary Table S2 (indirect effect 1 = 0.10, *p* < 0.001; indirect effect 2 = 0.15, *p* < 0.001; indirect effect 3 = 0.18, *p* < 0.001; indirect effect 7 = 0.08, *p* < 0.01; indirect effect 8 = 0.11, *p* < 0.01; indirect effect 9 = 0.14, *p* < 0.01). On the other hand, PE emotions are confirmed as a full mediator only in the relationship between PE with others and PE intensity (indirect effect 12 = 0.03, *p* < 0.05). The rest of the mediations through PE emotions did not occur since the indirect effects were not significant. All given mediations are full since all the direct effects are not significant (see Supplementary Table S2).

## 3 Study 3

In Study 2, we proposed an intra-individual analysis to observe whether daily variations in the frequency, intensity, and duration of PE are positively associated with PE-related emotions on a daily basis. Furthermore, we examined the role of prioritizing positivity as a vantage resource that enhances the effectiveness of PE and its effects on PE-related emotions.

So far, following the Upward Spiral Theory of lifestyle Change (9), the hypothesized model was tested with the following hypotheses and is depicted in Supplementary Figure S2:

○ Hypothesis 1: Different characteristics of PE, such as its frequency, duration, and intensity, will be positively associated with PE emotions.
○ Hypothesis 2: PE-related emotion will be positively associated with PE characteristics (i.e., frequency, duration, and intensity).
○ Hypothesis 3: PE-related emotions will be positively associated with prioritizing positivity.
○ Hypothesis 4: Prioritizing positivity will modulate the relationship between PE characteristics (frequency, duration, and intensity) and PE-related emotions.

### 3.1 Materials and methods: study 2

#### 3.1.1 Participants and protocols

For study 2, we invited voluntary participants from study 1 to join a diary study, where they would have to fill out a questionnaire three times a day (M1, before work/in the morning; M2, after work/in the afternoon; M3, in the evening) for a full week (Monday to Sunday). Of the 1,266 people who answered the first questionnaire, 343 agreed to participate in Study 2. During this phase, COVID-19 restrictions were still in place, but people were allowed to leave their homes. We began Phase 2 by emailing 314 participants with details about the study and links to the questionnaires. In this email, they were also given the option of having the researchers send out reminders at each moment of every day. Participants who chose this option could choose to be part of an instant messaging group or to be notified by email. During the week of the study, daily reminders were sent to the people who requested it, in addition to a mass mail in the middle of the week, encouraging their participation. To stimulate participation, 40 checks of 40€ each were also raffled among the participants who reached the end of the study.

Finally, of the 314 subjects we initially contacted, we were left with a sample of 146 participants, according to the following criteria, with the second criterion applied to the result of the first:

1. They had responded at least four full days or 16 moments throughout the entire week (76% of the total moments).
2. Answers at each moment of the day were separated by a minimum of 15 min.

Of these 146 participants, 77% were women, with a mean age of 34.8 years (SD = 13); In addition, 49.3% worked during confinement, with 42% of them working from home.

#### 3.1.2 Measures

The variables and questionnaires used for the study are described as follows:

• PE characteristics were evaluated using three indicators, and the hypothesized model was tested separately for each: first, the frequency with which the participants carried out physical exercise during the week (from once a week to more than once a day); second, the duration of the physical exercise session in minutes; and third, the intensity level of the physical exercise session, which was measured using a six-point single-item visual analog scale (19, 20), asking them to indicate the battery level that best represented the intensity during their physical exercise sessions (1 = almost empty battery/low intensity and 6 = full battery/maximum intensity) (mean = 3.87; SD = 1.15).
• PE emotions were measured using a 7-point visual analog scale (19, 20), where a single item asked them to indicate the face that best expressed their emotional wellbeing that day (0 = sad face and 6 = happy face).
• Prioritizing positivity was measured through a three-item scale (17) (α = 0.92), (“A priority for me today has been experiencing happiness”; “Today, I have sought and nurtured my positive emotions”; “Today, I have structured my day to maximize my happiness”). It was measured with a Likert-type scale ranging from 0 (never) to 6 (always). These items were only included in the third measurement of the day.

#### 3.1.3 Data analysis

Descriptive statistics, including means, standard deviations, correlations, and Cronbach's alpha, are presented in Supplementary Table S3. Prior to conducting further analysis and hypothesis testing, we calculated the intra-class correlation coefficient (ICC) to examine the between-person and within-person variance in day-level variables.

The between-person variance was 45.41% for PE emotions and 60.52% for prioritizing positivity. Thus, our variables exhibited both between- and within-person variance, warranting further examination of predictors at the person and day levels.

To test all four hypotheses, we followed the same procedure, utilizing multilevel analysis in MLwin 2.32 software (27). Following the recommendations of Ohly et al. (28), all day-level variables were person-centered.

First, for Hypotheses 1 and 4, we tested a null or intercept-only model. Next, we introduced control variables in Model 1, namely, gender as a categorical value and day number, to test the potential growth effects of PE emotions during the week. We did this as a strategy to capture “contaminating” variables that could bias the results. Specifically, there is previous research that indicates that levels of PE emotions vary depending on the day of the week (29, 30), and PE depending on gender (31). In Model 2, we introduced the main effect variables for the different hypotheses. Finally, in Model 3, we tested for the interaction effect of prioritizing positivity and PE characteristics, mentioned in Hypothesis 4.

For Hypotheses 2 and 3, we ran four separate equations for each of the PE characteristics and prioritizing positivity as dependent variables. We started with a null or intercept-only model, followed by Model 1, which introduced control variables such as gender and day of the week. Finally, in Model 2, we tested for the main effects, specifically introducing PE-related exercise as a predictor.

### 3.2 Study 2 results

Hypothesis 1 proposed that PE characteristics (frequency, duration, and intensity) will be positively associated with PE emotions. As shown in Supplementary Table S4, daily PE frequency (*β* = 0.18, SE = 0.09, *p* = 0.038) and intensity (*β* = 0.21, SE = 0.03, *p* = 0.001) were significant predictors of PE emotions. On the contrary, PE duration was not a significant predictor (*β* = 0.01, SE = −0.03, *p* = 0.92). Thus, Hypothesis 1 is only partially supported since two out of three PE characteristics showed a positive association with PE emotions. Regarding control variables, in the final model (Model 3) that included interaction terms between prioritizing positivity and PE characteristics, neither gender (*β* = 0.22, SE = 0.142, *p* = 0.17) nor day of the week (*β* = −0.02, SE = 0.02, *p* = 0.25) was a significant predictor.

Hypothesis 2 proposed that PE emotions will be positively associated with PE characteristics (frequency, duration, and intensity) as a predictor. Supplementary Tables S5–S7 present the results of the models tested for each of the PE characteristics as dependent variables. For PE frequency, the relation with PE emotions was not significant (*β* = 0.03, SE = 0.02, *p* = 0.17). For PE duration and intensity, the relation with PE emotions was significant in both cases (duration: *β* = 0.21, SE = 0.06, *p* = 0.035; intensity: *β* = 0.39, SE = 0.05, *p* = 0.004). Therefore, Hypothesis 2 is partially supported since PE emotions were a significant predictor of only two PE characteristics, namely, duration and intensity. Among the control variables, day of the week was the only significant predictor of PE duration (*β* = 0.09, SE = 0.02, *p* = 0.021), while the rest of the relations were non-significant.

Hypothesis 3 proposed that PE emotions were positively associated with prioritizing positivity. Results shown in Supplementary Table S8 indicate the PE emotions were a significant predictor of prioritizing positivity (*β* = 0.35, SE = 0.05, *p* = 0.005). Among the control variables, day of the week was the only significant predictor (*β* = 0.10, SE = 0.02, *p* = 0.007). Therefore, Hypothesis 3 is supported.

Finally, Hypothesis 4 proposed that prioritizing positivity modulated the relationship between PE characteristics (frequency, duration, and intensity) and PE emotions. Results presented in Supplementary Table S4 indicate that prioritizing positivity was a significant predictor of PE-related emotions (*β* = 0.32, SE = 0.08, *p* = 0.004). As per the interaction terms, we tested for the interaction between prioritizing positivity and frequency and intensity. Since PE duration was not a significant predictor in the first place, we excluded it from further analyses. The interaction terms for both PE frequency (*β* = 0.06, SE = 0.04, *p* = 0.143) and intensity (*β* = −0.03, SE = 0.02, *p* = 0.109) were not significant. Therefore, Hypothesis 4 is not supported.

## 4 Discussion

This study examined the mechanisms that help PE become a recurring habit using two approaches (between-subjects and within-subject) based on the Upward Spiral Theory of Lifestyle Change (9). On the one hand, we conducted a between-subjects study (Study 1) to know whether individuals who prioritized positivity experienced more positive emotions when performing PE and, in turn, whether these individuals performed PE more frequently, for longer sessions, and with higher intensity. On the other hand, from a within-subject perspective, we conducted another study (Study 2) to test whether daily variations in the frequency, intensity, and duration of PE were positively associated with PE-related emotions. Furthermore, we examined the role of prioritizing positivity as a vantage resource that improves the effectiveness of PE and its effects on PE emotions. The results we obtained from both studies were mixed.

### 4.1 Theoretical and practical implications

The evidence from Study 1 suggests that the mechanism that promotes more frequent, longer, and higher-intensity PE sessions is driven by positive psychological constructs such as engagement and positive emotions experienced doing physical exercise. Even more, it occurs when individuals are doing PE with others and prioritize positivity organizing their day to include behaviors that are a source of positive emotions. In line with previous research on cognitive appraisals and their impact on perceived self-efficacy and distress during challenging periods, such as the COVID-19 lockdown (32), our findings also highlight the significant role of psychological factors in influencing physical exercise behaviors and wellbeing. However, the psychological mechanisms involved in these processes differ when it comes to PE engagement vs. positive emotions. On the one hand, when PE engagement is the psychological mechanism that explains these relationships, individuals who prioritize positivity experience higher levels of engagement when performing PE, which, in turn, leads to more frequent, longer, and more intense PE sessions. The same thing happens when doing PE with others—a greater sense of engagement in P, leads to increased frequency, duration, and intensity of the PE. On the other hand, when emotions serve as the psychological mechanism, only performing PE with others acts as the driver, which also leads to (only) higher PE intensity. So far, it seems that engagement in physical exercise is the main psychological mechanism that explains how prioritizing positivity and doing PE with others influence the frequency, duration, and intensity of the physical activity. Anyway, it is interesting to highlight that when people are doing physical exercise with others, they not only experience greater engagement but also positive emotions that influence their exercise-related physical behaviors.

This leads us to think that for the PE sessions to become more frequent, intense, and longer in duration, the positive psychological experience when performing PE should be rather eudaimonic (i.e., engagement in the PE) than hedonic (i.e., positive emotions).

Furthermore, Study 2 focused on daily variations in the relationship between prioritizing positivity and other variables from a within-person perspective. We focused on variations of PE frequency, duration, and intensity as predictors of PE-related emotions and vice versa. As well, we looked at how prioritizing positivity as a vantage resource could potentially amplify the effect of physical exercise on PE emotions. The results showed that the relation between PE intensity with PE-related emotions was reciprocal and positive over time. Similar results were obtained in Study 1, where emotions were only related to the PE intensity (rather than frequency and duration). This suggests that daily PE intensity could play a more prominent role compared to PE duration and frequency.

Regarding the modulating role of prioritizing positivity, it did not moderate the relationship between PE characteristics and PE emotions. The explanation for these partial results could be that a daily study may not capture the changes required for a change in habit since these potentially need more time to become a habit. Regarding prioritizing positivity as a vantage resource, the explanation can go along the same lines, understanding that prioritization tends to be more stable over time, more characteristic, and, therefore, more difficult to capture in a daily study.

However, prioritizing positivity was positively and reciprocally associated with PE emotions, showing its role as a driving mechanism rather than a moderator. This shows the recurrence between emotions and prioritization positivity. Creating a loop that begins with the behavior (PE) that promotes positive emotions, which, in turn, promotes prioritizing the positive, which influences the emotion again. In other words, a linking positive cycle of affective and behavioral wellbeing is created (a positive spiral). This leads us to rethink the theoretical model, where instead of asking under what conditions vantage resources moderate the link between PE and emotions, we can ask how these same resources could predict and link the psychological process among PE, emotions, and resources.

Anyway, our results confirmed the hypotheses regarding prioritizing positivity, described by Fredrickson et al. (17), who found that individuals who prioritize positive activities in their lives experience better psychological wellbeing, greater life satisfaction, and more engagement in their activities, similar to what we observed with PE in our study. In this way, teaching people how to prioritize positivity in their lives, for example, by implementing positive psychological interventions based on goal setting (33) and life crafting (34), will affect their ability to prioritize positivity and engage more effectively and positively in activities like doing physical exercise and abandoning a sedentary lifestyle.

### 4.2 Study limitations and future research

Despite the strengths of our studies, we acknowledge several limitations. The first limitation is that the studies were carried out in a very extreme and unusual context, such as pandemic confinement. Emotions could have been affected by psychological factors (e.g., fatigue, depression, chronic stress, and languishing) or physical factors (e.g., physical limitations, uncomfortably built environments, and limited time), which may have been more unexpected and abrupt during the confinement and were not considered in our research (35–38). Therefore, it would be beneficial to repeat the studies in a more normalized context while controlling for these variables.

A second limitation relates to Study 2 (the diary study), where we aimed to determine how today's feelings influence the repetition of tomorrow’s behavior. However, we can have a behavior that is established as a habit in life, but maybe this behavior is generated by other causes, such as family obligations, rather than what that behavior generates.

A third and final limitation relates to the variable of prioritizing positivity. This limitation is related to the previous limitation, as it asks about prioritizing positivity levels in general. This implies that when participants report prioritizing positivity, they may not necessarily be prioritizing PE but focusing on other activities that provide positive emotions, and on that day, PE might not be one of them. Therefore, in future studies, framing the question specifically around prioritizing positivity in relation to PE could help address this limitation, as well as partially address the previous limitation.

## 5 Conclusions

This study aimed to understand the psychological mechanisms that help physical exercise become a habit. On the one hand, a cross-sectional study demonstrated that resources such as prioritizing positivity and PE with others are good drivers of higher PE frequency, intensity, and even longer sessions, provided these behaviors are mediated by PE engagement, which involves a genuine and stable commitment to PE. Since something more ephemeral, like PE-related emotions, only predicts a more intense PE and not more frequent or long-lasting sessions, the diary study revealed additional insights. We found two loops, established by the Upward Spiral Theory of Lifestyle Change (9), but these loops did not occur since prioritizing positivity does not modulate the relationship between PE and PE-related emotions. However, we were able to observe a spiral in which a concatenation of recursive effects was produced: behavior, emotion, prioritization and emotion, and back to behavior. Furthermore, this recursive spiral was found only with PE intensity, not with frequency or duration. We found that this is in line with a cross-sectional study, where emotions only influence the PE intensity rather than the rest of the characteristics of the PE, such as frequency and duration.

## Data Availability

The raw data supporting the conclusions of this article will be made available by the authors without undue reservation.
