# Supplementary material for: How physical exercise with others and prioritizing positivity contribute to (work) wellbeing: a cross-sectional and diary multilevel study
Source: Front Sports Act Living. 2024 Sep 6;6:1437974. doi: 10.3389/fspor.2024.1437974 (PMC11412860; doi:10.3389/fspor.2024.1437974)
Supplement: Supplementary file 1 [file Datasheet1.docx]

Supplementary Material

# Supplementary Figures and Tables

## Supplementary Figures


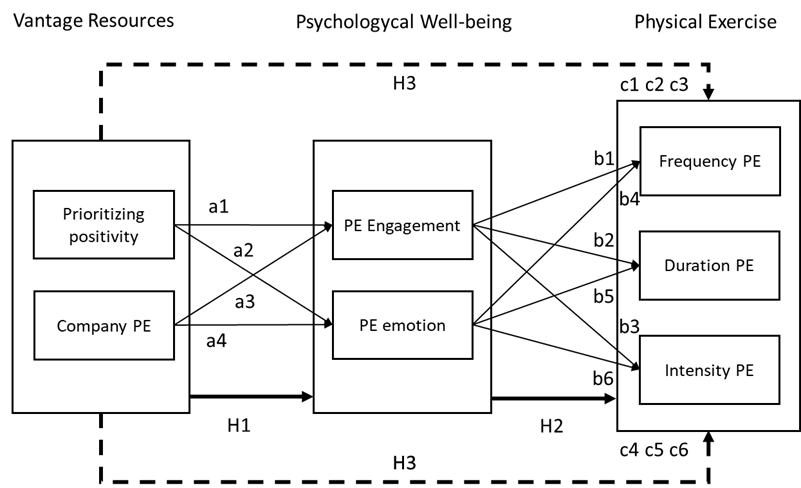


**Supplementary Figure 1.** The Hypothesized Model Study 1. *Note*: PE = Physical exercise.


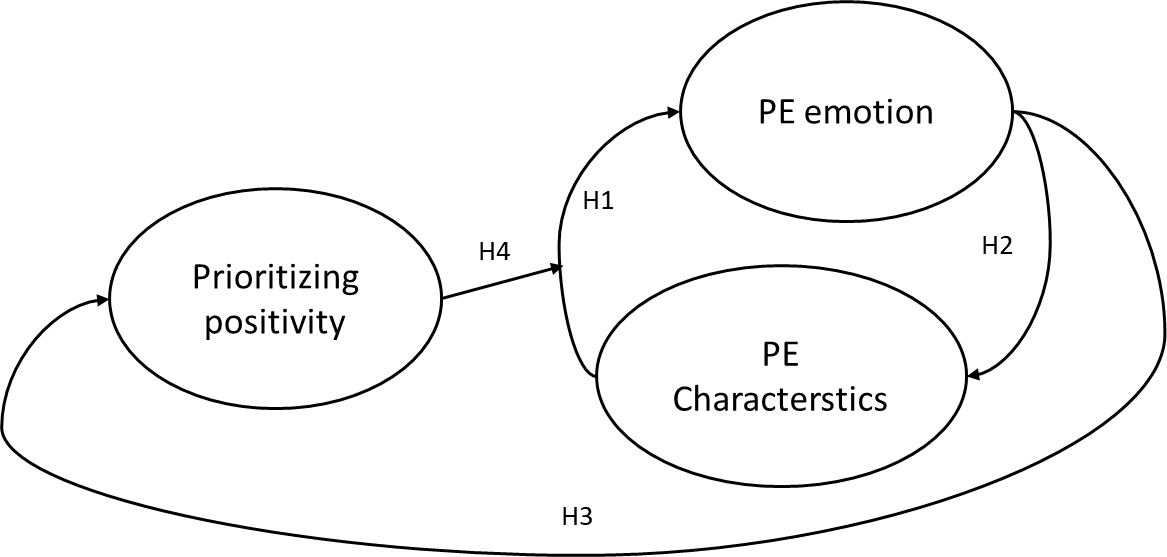


**Supplementary Figure 2.** Hypothesized Model Study 2. *Note*: PE = Physical exercise.

## Supplementary Tables

**Table 1**

Descriptive Statistics and Correlations between the Study Variables.

|  |  | **Variables** | ***M*** | ***SD*** | **1** | **2** | **3** | **4** | **5** | **6** | **7** |
| --- | --- | --- | --- | --- | --- | --- | --- | --- | --- | --- | --- |
|  | 1 | Prioritizing positivity | 4.05 | 1.01 |  |  |  |  |  |  |  |
|  | 2 | PE in Company |  |  | -.01 |  |  |  |  |  |  |
|  | 3 | PE emotion | 5.06 | 0.88 | .31** | .09* |  |  |  |  |  |
|  | 4 | PE engagement | 4.33 | 1.08 | .34** | .12** | .72** |  |  |  |  |
|  | 5 | PE frequency | 2.44 | 1.11 | .09* | -.03 | .24** | .30** |  |  |  |
|  | 6 | PE duration | 1.46 | 1.12 | .10* | -.01 | .29** | .39** | .30** |  |  |
|  | 7 | PE intensity | 2.91 | 1.24 | .15** | -.07 | .40** | .48** | .18** | .43** |  |
| *Note*. The correlation is significant at the level of * p < .05 and ** p < .01 (bilateral). M = Means; SD = Standard Deviations; PE = Physical Exercise | | | | | | | | | | | |

**Table 2**

Mediation Model.

|  |  | Estimate *β* | *SE* | Hypotheses |
| --- | --- | --- | --- | --- |
| Path a1: Prioritizing positivity --> PE Engagement |  | .37 | .04*** | 1 |
| Path a2: Prioritizing positivity --> PE emotions |  | .27 | .04*** | 1 |
| Path a3: Company --> PE Engagement |  | .29 | .09** | 1 |
| Path a4: Company --> PE emotions |  | .18 | .08* | 1 |
| Path b1: PE Engagement --> Frequency PE |  | .28 | .05*** | 2 |
| Path b2: PE Engagement --> Duration PE |  | .41 | .04*** | 2 |
| Path b3: PE Engagement --> Intensity PE |  | .48 | .05*** | 2 |
| Path b4: PE emotions --> Frequency PE |  | .06 | .06 | 2 |
| Path b5: PE emotions --> Duration PE |  | .03 | .05 | 2 |
| Path b6: PE emotions --> Intensity PE |  | .15 | .06* | 2 |
| Path c1: Prioritizing positivity --> Frequency PE |  | -.02 | .05 | 3 |
| Path c2: Prioritizing positivity --> Duration PE |  | -.05 | .05 | 3 |
| Path c3: Prioritizing positivity --> Intensity PE |  | -.04 | .05 | 3 |
| Path c4: Company --> Frequency PE |  | -.03 | .10 | 3 |
| Path c5: Company --> Duration PE |  | -.15 | .10 | 3 |
| Path c6: Company --> Intensity PE |  | .02 | .10 | 3 |
| Prioritizing positivity <--> Company |  | -.00 | .02 |  |
| Indirect effect 1: a1 x b1 Prioritizing positivity --> PE engagement --> Frequency PE | | .10 | .09*** | 3 |
| Indirect effect 2: a1 x b2 Prioritizing positivity --> PE engagement --> Duration PE | | .15 | .13*** | 3 |
| Indirect effect 3: a1 x b3 Prioritizing positivity --> PE engagement --> Intensity PE | | .18 | .15*** | 3 |
| Indirect effect 4: a2 x b4 Prioritizing positivity --> PE emotions --> Frequency PE | | .02 | .02 | 3 |
| Indirect effect 5: a2 x b5 Prioritizing positivity --> PE emotions --> Duration PE | | .01 | .01 | 3 |
| Indirect effect 6: a2 x b6 Prioritizing positivity --> PE emotions --> Intensity PE | | .04 | .04 | 3 |
| Indirect effect 7: a3 x b1 Company --> PE engagement --> Frequency PE | | .08 | .03** | 3 |
| Indirect effect 8: a3 x b2 Company --> PE engagement --> Duration PE | | .11 | .05** | 3 |
| Indirect effect 9: a3 x b3 Company --> PE engagement --> Intensity PE | | .14 | .05** | 3 |
| Indirect effect 10: a4 x b4 Company --> PE emotions --> Frequency PE | | .01 | .01 | 3 |
| Indirect effect 11: a4 x b5 Company --> PE emotions --> Duration PE | | .01 | .00 | 3 |
| Indirect effect 12: a4 x b6 Company --> PE emotions --> Intensity PE | | .03 | .01* | 3 |

*Note*. * *p* < .05, ** *p* < .01 and *** *p* < .001 (bilateral). PE = Physical Exercise

**Table 3**

Descriptive Statistics and Correlations between the Study 2 Variables.

|  | M (SD) | 1 | 2 | 3 | 4 | 5 | 6 | 7 | 8 |
| --- | --- | --- | --- | --- | --- | --- | --- | --- | --- |
| Age | 34.70 (12.96) | - | - | - | - | - | - | - | - |
| Gender |  | -.237** | - | - | - | - | - | - | - |
| Day | 4.00 (1.99) | -.003 | .0 | - | - | - | - | - | - |
| PE frequency | .64 (.61) | .100** | .010 | -.026 | - | - | - | - | - |
| PE duration | 1.92 (1.35) | -.047 | -.013 | .153** | .210** | - | - | - | - |
| PE intensity | 3.87 (1.15) | -.032 | -.094* | .004 | .011 | .315** | - | - | - |
| Prioritizing positivity | 3.73 (1.30) | -.108** | .238** | .136** | .106** | .142** | .179** | - | - |
| PriPos*PE frequency | 2.34 (2.61) | .058 | .073* | .066* | .903** | .204** | .147** | .428** | - |
| PriPos*PE intensity | 15.17 (7.53) | -.109* | .069 | .098* | .048 | .289** | .737** | .783** | .530** |
| *Note*. * *p* < .01. ** *p* < .001. M = Means; SD = Standard Deviations; PE = Physical Exercise; PriPos = Prioritizing Positivity | | | | | | | | | |

**Table 4**

Multilevel Estimates for Models Predicting **PE Emotion**, N = 146 Participants, N = 1,022 Data Points.

|  | **Model 1** | | | | | **Model 2** | | | **Model 3** | | | | |  |  |
| --- | --- | --- | --- | --- | --- | --- | --- | --- | --- | --- | --- | --- | --- | --- | --- |
|  | **Estimate** | **SE** | ***p*** | | **Estimate** | | **SE** | ***p*** | **Estimate** | **SE** | ***p*** | |  |  |  |
| Constant | 4.580 | .063 | *** | | 4.453 | | .137 | *** | 4.598 | .141 | *** | |  |  |  |
| Day | .013 | .015 | ns | | .006 | | .015 | ns | -.019 | .015 | ns | |  |  |  |
| Gender (0 = male, 1 = female) | .198 | .150 | ns | | .270 | | .137 | ns | .223 | .142 | ns | |  |  |  |
| PE frequency |  |  |  | | .184 | | .090 | * | -.022 | .157 | ns | |  |  |  |
| PE intensity |  |  |  | | .208 | | .033 | ** | .295 | .055 | ** | |  |  |  |
| PE duration |  |  |  | | -.002 | | .033 | ns | .000 | .032 | ns | |  |  |  |
| Prioritizing positivity |  |  |  | | .262 | | .036 | ** | .320 | .084 | ** | |  |  |  |
| PE frequency * Prioritizing positivity |  |  |  | |  | |  |  | .060 | .037 | ns | |  |  |  |
| PE intensity * Prioritizing positivity |  |  |  | |  | |  |  | -.027 | .015 | ns | |  |  |  |
| -2*log | 1440.440 | | |  | | 1203.607 | |  | 1210.918 | | |  | |  |  |
| Diff-2*log | 17.48 | | | *** | | 236.833 | | *** | 7.311 | | | ns | |  |  |
| d.f. | 4 | | |  | | 6 | |  | 8 | | |  | |  |  |
| Between-person (Level 2) variance (SE) | .394 | .064 |  | | .308 | | .052 |  | .334 | .055 |  | |  |  |  |
| Within-person (Level 1) variance (SE) | .486 | .032 |  | | .406 | | .029 |  | .405 | .029 |  | |  |  |  |
| *Note*. * *p* < .05. ** *p* < .01. *** *p* < .001. Model 1 was compared to a Null Model with the intercept-only (γ = 4.779; SE = .063; t = 75.857; -2*log = 1457.920; Level 1 Variance = .488; SE = .032). | | | | | | | | | | | | | | |  |
|  |  |  |  |  |  |  |  |  |  |  |  |  |  |  |  |

**Table 5**

Multilevel Estimates for Models Predicting **PE Frequency**, N = 146 Participants, N = 1,022 Data Points

|  | **Model 1** | | | **Model 2** | | | **Model 3** | | |
| --- | --- | --- | --- | --- | --- | --- | --- | --- | --- |
|  | **Estimate** | **SE** | ***p*** | **Estimate** | **SE** | ***p*** | **Estimate** | **SE** | ***p*** |
| Constant | .640 | .030 | *** | .566 | .070 | *** | .961 | .052 | *** |
| Day |  |  |  | -.008 | .009 | ns | .008 | .007 | ns |
| Gender (0 = male, 1 = female) |  |  |  | .138 | .070 | ns | .119 | .051 | ns |
| PE emotions |  |  |  |  |  |  | .032 | .018 | ns |
| -2*log | 1787.600 | |  | 1772.509 | |  | 483.948 | |  |
| Diff-2*log |  | |  | 15.091 | | ** | 1288.561 | |  |
| d.f. | 0 | |  | 2 | |  | 3 | |  |
| Between-person (Level 2) variance (SE) | .086 | .015 |  | .083 | .015 |  | .030 | .007 |  |
| Within-person (Level 1) variance (SE) | .291 | .014 |  | .291 | .014 |  | .112 | .007 |  |
| Notes: * *p* < .05. ** *p* < .01. *** *p* < .001. | | | | | | | | | |

**Table 6**

Multilevel Estimates for Models Predicting **PE Duration**, N = 146 Participants, N = 1022 Data Points

|  | **Model 0** | | | **Model 1** | | | **Model 2** | | |
| --- | --- | --- | --- | --- | --- | --- | --- | --- | --- |
|  | **Estimate** | **SE** | ***p*** | **Estimate** | **SE** | ***p*** | **Estimate** | **SE** | ***p*** |
| Constant | 1.834 | .087 | *** | 1.608 | .200 | *** | 1.646 | .198 | *** |
| Day |  |  |  | .093 | .021 | ** | .093 | .021 | ** |
| Gender (0 = male, 1 = female) |  |  |  | -.164 | .207 | ns | -.205 | .206 | ns |
| PE emotions |  |  |  |  |  |  | .213 | .058 | * |
| -2*log | 1875.667 | |  | 1842.039 | |  | 1824.314 | |  |
| Diff-2*log |  | |  | 33.628 | | *** | 17.725 | | ** |
| d.f. | 0 | |  | 2 | |  | 3 | |  |
| Between-person (Level 2) variance (SE) | .761 | .124 |  | .746 | .122 |  | .735 | .120 |  |
| Within-person (Level 1) variance (SE) | .999 | .066 |  | .967 | .064 |  | .941 | .063 |  |
| Note: * *p* < .05. ** *p* < .01. *** *p* < .001. | | | | | | | | | |

**Table 7**

*Multilevel Estimates for Models Predicting PE Intensity, N = 146 Participants, N = 1,022 Data Points*.

|  | **Model 0** | | | **Model 1** | | | **Model 2** | | |
| --- | --- | --- | --- | --- | --- | --- | --- | --- | --- |
|  | **Estimate** | **SE** | ***p*** | **Estimate** | **SE** | ***p*** | **Estimate** | **SE** | ***p*** |
| Constant | 3.826 | .077 | *** | 3.860 | .180 | *** | 3.935 | .166 | *** |
| Day |  |  |  | .010 | .019 | ns | .010 | .018 | ns |
| Gender (0 = male, 1 = female) |  |  |  | -.095 | .187 | ns | -.180 | .170 | ns |
| PE emotions |  |  |  |  |  |  | .389 | .050 | ** |
| -2*log | 1721.377 | |  | 1710.287 | |  | 1648.691 | |  |
| Diff-2*log |  | |  | 11.09 | | ** | 61.596 | | *** |
| d.f. | 0 | |  | 2 | |  | 3 | |  |
| Between-person (Level 2) variance (SE) | .608 | .098 |  | .612 | .099 |  | .485 | .082 |  |
| Within-person (Level 1) variance (SE) | .769 | .051 |  | .773 | .051 |  | .719 | .048 |  |
| *Note*. * *p* < .05. ** *p* < .01. *** *p* < .001. | | | | | | | | | |

**Table 8**

Multilevel Estimates for Models Predicting **Prioritizing Positivity**, N = 146 Participants, N = 1,022 Data Points.

|  | **Model 0** | | | **Model 1** | | | **Model 2** | | | |
| --- | --- | --- | --- | --- | --- | --- | --- | --- | --- | --- |
|  | **Estimate** | **SE** | ***p*** | **Estimate** | **SE** | ***p*** | **Estimate** | **SE** | ***p*** | |
| Constant | 3.715 | .090 | *** | 3.554 | .189 | *** | 3.672 | .195 | *** | |
| Day |  |  |  | .107 | .014 | ** | .097 | .015 | ** | |
| Gender ( 0 = male, 1 = female) |  |  |  | -.338 | .203 | ns | -.244 | .210 | ns | |
| PE emotions |  |  |  |  |  |  | .352 | .046 | ** | |
| -2*log | 2349.145 | |  | 2272.879 | |  | 1339.183 | |  | |
| Diff-2*log | - | |  | 76.266 | | *** | 933.696 | | *** | |
| d.f. | 0 | |  | 2 | |  | 3 | |  | |
| Between-person (Level 2) variance (SE) | 1.038 | .138 |  | .985 | .129 |  | .904 | .125 |  | |
| Within-person (Level 1) variance (SE) | .677 | .036 |  | .625 | .034 |  | .422 | .030 |  | |
| *Note*. * p < .05. ** p < .01. *** p < .001. | | | | | | | | | |  |
